# Development and Validation of a Food Frequency Questionnaire to Estimate Intake among Children and Adolescents in Urban Peru

**DOI:** 10.3390/nu9101121

**Published:** 2017-10-14

**Authors:** Carly A. Rodriguez, Emily R. Smith, Eduardo Villamor, Nelly Zavaleta, Graciela Respicio-Torres, Carmen Contreras, Sara Perea, Judith Jimenez, Karen Tintaya, Leonid Lecca, Megan B. Murray, Molly F. Franke

**Affiliations:** 1 Department of Global Health and Social Medicine, Harvard Medical School, Boston, MA 02115, USA; carly_rodriguez@hms.harvard.edu (C.A.R.); llecca_ses@pih.org (L.L.); megan_murray@hms.harvard.edu (M.B.M.); 2 Division of Gastroenterology, Hepatology and Nutrition, Boston Children’s Hospital, Boston, MA 02115, USA; ersmith@hsph.harvard.edu; 3 Department of Global Health and Population, Harvard TH Chan School of Public Health, Boston, MA 02115, USA; 4 Department of Epidemiology, School of Public Health, University of Michigan, Ann Arbor, MI 48109, USA; villamor@umich.edu; 5 Instituto de Investigación Nutricional, Ave La Molina 1885, La Molina, Lima 12, Peru; nzavaleta@ins.gob.pe (N.Z.); grespicio@hotmail.com (G.R.T.); 6 Socios En Salud Sucursal Peru, Ave Merino Reyna 575, Carabayllo, Lima 6, Peru; ccontreras_ses@pih.org (C.C.); sperea_ses@pih.org (S.P.) jjimenez_ses@pih.org (J.J.); ktintaya_ses@pih.org (K.T.)

**Keywords:** food frequency questionnaire, validation, reproducibility

## Abstract

Tools to assess intake among children in Latin America are limited. We developed and assessed the reproducibility and validity of a semi-quantitative food frequency questionnaire (FFQ) administered to children, adolescents, and their caregivers in Lima, Peru. We conducted 24-h diet recalls (DRs) and focus groups to develop a locally-tailored FFQ prototype for children aged 0–14 years. To validate the FFQ, we administered two FFQs and three DRs to children and/or their caregivers (*N* = 120) over six months. We examined FFQ reproducibility by quartile agreement and Pearson correlation coefficients, and validity by quartile agreement and correlation with DRs. For reproducibility, quartile agreement ranged from 60–77% with correlations highest for vitamins A and C (0.31). Age-adjusted correlations for the mean DR and the second-administered FFQ were highest in the 0–7 age group, in which the majority of caregivers completed the FFQ on behalf of the child (total fat; 0.67) and in the 8–14 age group, in which both the child and caregiver completed the FFQ together (calcium, niacin; 0.54); correlations were <0.10 for most nutrients in the 8–14 age group in which the caregiver completed the FFQ on the child’s behalf. The FFQ was reproducible and the first developed and validated to assess various nutrients in children and adolescents in Peru.

## 1. Introduction

A significant proportion of disease and death can be attributed to suboptimal nutrition. Poor nutrition is thought to be the underlying cause of most infectious illness in children and ultimately responsible for a substantial proportion of child mortality globally [1]. When considering that nutrition is an important modifiable risk factor, reliable assessment of diet in children and adolescents—who are at a unique point for early intervention—is a major research priority [2,3]. Dietary assessment is critical to informing disease prevention efforts and understanding the complex relationship between diet and disease.

Assessment of intake relies on validation of tools designed for and implemented in a target population. One method of assessing intake is the food frequency questionnaire (FFQ), consisting of a finite list of food items for which respondents indicate the frequency of consumption. FFQs must be developed and validated for a specific population due to socioeconomic, cultural, geographic, and seasonal differences that can vary substantially between and within countries [4,5]. FFQs are both practical and efficient to administer, making them a popular assessment method in research studies involving long-term diet; however, few FFQs have been designed or validated for use in children and adolescents, especially in Latin America [6,7,8]. Among three systematic reviews of dietary assessment tools for children representing over 30 studies, only three were in low- or middle-income countries, including two from Brazil and one from Uganda [6,7,8]. The lack of tools for children and adolescents validated in a variety of settings may be due to challenges in assessing intake in a group with rapidly changing dietary patterns.

We developed and implemented a semi-quantitative FFQ designed for children and adolescents living in urban Peru and assessed the FFQ’s reproducibility and relative validity as compared to 24-h diet recalls (DR).

## 2. Materials and Methods

### 2.1. Study Participants

#### 2.1.1. Food Frequency Questionnaire Development

We recruited adult caregivers who participated in FFQ development from those seeking immunizations and child growth services at local hospitals and health centers of the Peruvian Ministry of Health. These local hospitals and health centers are where the majority of the urban population in Lima, Peru seek care. In preliminary DRs, we enrolled 60 caregivers who spent the majority of the day caring for their child and were most knowledgeable on their child’s daily intake. Preliminary DR participants were coincidentally all female. For focus groups, we recruited 48 female adult participants (six focus groups of eight participants) who had primary responsibility for cooking in their respective households. We restricted the focus groups to female adults because evidence suggests that women are largely responsible for household food purchases and preparation in Peru, and are thus the most knowledgeable on household diet [9]. For the pilot test of the FFQ prototype, we enrolled 20 female caregivers who spent the majority of the day caring for their child.

#### 2.1.2. Food Frequency Questionnaire Validation

We recruited a convenience sample of 120 participants who consented to being contacted for future studies from a population-based prospective cohort study of over 14,000 household contacts of adults with tuberculosis in Lima, which has been described previously [10,11,12,13]. These participants sought care at the same health centers as participants from the FFQ development. While participants in the FFQ development phase and validation study differ in that the latter came from households with a known tuberculosis case, we believe these populations are generally similar because all of the study participants in both phases were seeking care at the same health centers. Participants were enrolled in the FFQ validation study if they were under 15 years of age, did not have a diagnosis of tuberculosis disease, agreed to provide a blood specimen, and had a parent or guardian capable of recalling dietary intake on behalf of, or in collaboration with, the participant. At the time of enrollment, we provided consent forms in Spanish to each participant’s legal parent or guardian describing study procedures and risks in detail. Additionally, we sought informed assent from children ages 8–14 years. The Institutional Review Board of Harvard TH Chan School of Public Health and the Research Ethics Committee of the National Institute of Health of Peru approved the study protocol.

### 2.2. Data Collection

#### 2.2.1. Anthropometric Assessment

We used a calibrated spring scale in children under two years of age and a floor scale in those two and older to obtain weight information. We measured length in children under two years with an infantometer and height in children two and older with a stadiometer. Weight-for-age, length- or height-for-age, and weight-for-length Z-scores were calculated, as recommended by the US Centers for Disease Control and Prevention [14,15,16]. We used the 2006 World Health Organization growth charts for children aged less than 24 months and the 2000 US Centers for Disease Control and Prevention growth charts for children 24 months of age or greater.

#### 2.2.2. Food Frequency Questionnaire Development

We developed a semi-quantitative FFQ following the methods proposed by Willett [4]. First, we collected preliminary DRs from 60 caregivers and their children on randomly selected days, including weekdays and weekends. We interviewed children and their caregivers on the foods and beverages the child consumed within the previous 24-h period, using props including containers, serving utensils, and food models to illustrate portion size and to aid in recall. Next, we held six focus groups with a convenience sample of eight female volunteers each to assess the intake of specific regional and ethnic foods, typical portion sizes, seasonal variation in food availability, cooking methods, intake hierarchies within households, and other relevant issues that arose from the preliminary DRs. Based on findings from the preliminary DRs and focus groups, we developed the FFQ prototype following the structure of the Harvard semi-quantitative FFQ, which has previously been implemented in Latin America, including Mexico and Costa Rica [17,18]. We pilot tested the FFQ prototype in 20 female caregivers. Participants were asked to indicate the number of times per day, week, or month each item was consumed by selecting one of nine frequency options: never, once per month, one-three times per month, once per week, two-four times per week, five-six times per week, once per day, two-three times per day, and four-five times per day. We modified the prototype based on participant and interviewer feedback resulting in an FFQ with 150 food and beverage items, as well as questions on the frequency of vitamin or mineral intake and history of breastmilk consumption. Portion size was not included in the FFQ. Participants were asked to report on their intake in the previous year.

#### 2.2.3. Food Frequency Questionnaire Validation

**Figure 1:**
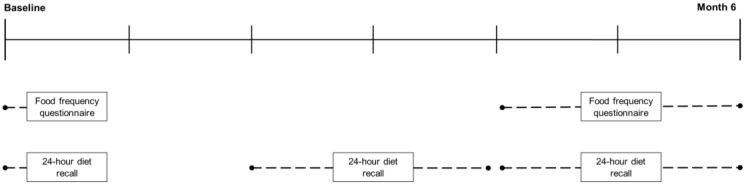
Implementation of study protocol to validate a food frequency questionnaire against 24-h diet recalls among children and adolescents in Lima, Peru.

We collected two FFQs over the course of six months (Figure 1). The first-administered FFQ was collected in June (during the winter in Peru) and the second-administered FFQ was collected in December and January (during the summer in Peru). Trained study staff administered the FFQs according to the same method described above. No participants in the validation study had previously participated in the development of the FFQ. Caregivers and their child were present for the FFQ interviews. We recorded when children participated in FFQ completion with their caregiver.

We collected three DRs over a six-month period (Figure 1) according to the same method described during FFQ development (Section 2.2.2). DRs were collected on randomly selected days, including weekdays and weekends, at health centers and the offices of Socios En Salud Sucursal Peru. Diet recalls were on the child’s diet but were completed with assistance from their caregiver who spent the most time with the child and was knowledgeable on daily intake.

### 2.3. Statistical Analysis

#### 2.3.1. Nutritional Values of FFQ

We used the Nutrition Data System for Research (NDSR) software (Version 2016, Nutrition Coordinating Center, University of Minnesota, Minneapolis, MN, USA) to determine nutritional content for the majority of items included in the FFQ. When nutritional values for regional and ethnic foods specific to Peru were not in the NDSR system, we obtained them from Peru’s Centro Nacional de Alimentación y Nutrición (CENAN) [19], however the CENAN database comprised only a subset of nutrients in NDSR: total energy, total protein, total fat, total carbohydrates, calcium, zinc, iron, β-carotene, retinol, vitamin A, riboflavin, niacin, and vitamin C. We imputed nutrients that were not included in the CENAN database to those of a similar food contained within NDSR. We calculated total nutrient intake per participant by multiplying the frequency of consumption by the nutritional content of a standard portion for each item (e.g., once a week = 0.14 × nutritional content of standard portion), and summed the nutrient intakes across each participant.

#### 2.3.2. Nutritional Values of DR

We used local food composition tables from Peru’s Instituto de Investigación Nutricional to determine the daily nutrient intake from the DR. These tables are based on food composition tables from CENAN [19]. Nutritional values for each food or beverage were modified based on recipe and cooking method. DR data were analyzed using the Statistical Package for Social Sciences, Version 15.0 (SPSS Inc., Chicago, IL, USA).

#### 2.3.3. Reproducibility

For the reproducibility analysis, we first assessed the distribution of each nutrient in the FFQ for normality by both visual inspection and skewness, and log transformed nutrients with skewness greater than one. All of the nutrients were adjusted for total energy intake using the residual method proposed by Willett [20]. We analyzed: the percent difference of nutrient means between FFQs; percentage of participants in the same or an adjacent nutrient quartile (e.g., from the third- to second- or fourth-quartile); and, the percentage of participants moving between extreme quartiles (e.g., from the first- to fourth-quartile and vice versa). Reliability was calculated by Pearson correlation coefficients.

#### 2.3.4. Validity

To determine relative validity, we compared the second-administered FFQ to the mean of the three DRs. Because the nutrient database used to score DRs contained only a subset of the nutrients reported in the NDSR database used to score FFQs, correlations with DRs were conducted on a subset of the nutrients reported in the FFQ. We log transformed nutrients from the DR that were not normally distributed by visual inspection and with a skewness of greater than one. We calculated the percentage of participants in the same or adjacent quartile, and moving between extreme quartiles. We calculated energy-adjusted Pearson correlation coefficients using the residual method. Because we did not assess portion size and energy was consistent across ages, we opted to calculate age-adjusted partial Pearson correlation coefficients in subsequent analyses.

Within-person (s^2^_w_) and between-person (s^2^_b_) variances were calculated for the three DRs, which were used to deattenuate age-adjusted Pearson correlation coefficients using the equation: $\sqrt{1}+\left[\left(s^{2}{}_{w}/s^{2}{}_{b}\right)/3\right]$. Evidence suggests an increase in cognitive ability to accurately conceptualize time and self-report food intake by age eight [21], thus we report stratified results for ages 0–7 and 8–14 years. We examined FFQ performance in subgroups defined by who completed the FFQ: by both the caregiver and child or the caregiver alone. Finally, out of concern for attenuation of correlation coefficients and misclassification of quartiles due to seasonal changes in intake, we conducted the validation analysis on the first-administered FFQ and the mean of the three DRs, and the second-administered FFQ and closest-administered DR. We analyzed the FFQ, and conducted the reproducibility and validation analyses using SAS, Version 9.3 (SAS Institute Inc., Cary, NC, USA).

## 3. Results

Table 1 shows characteristics of study participants at the time of the second-administered FFQ. Participants were a mean age of 7.59 years (±3.64 years) and 50% were male. FFQs were completed by the caregiver alone for 77% of participants.

### 3.1. FFQ Reproducibility

Participants reported a higher total energy intake in the first-administered FFQ than the second-administered FFQ (Table 2, Table S1). Crude Pearson correlations were highest for vitamin A, vitamin C, and total monounsaturated fatty acids (0.31), and were less than 0.20 for 21% of nutrients included in the analysis. Correlations decreased for all of the nutrients when we adjusted Pearson correlation coefficients for total energy (Table S2), which has been observed in other studies [22,23]. For the majority of nutrients, 65–75% of participants in the first-administered FFQ were classified into the same or moved to an adjacent quartile in the second-administered FFQ; 5–11% of participants moved from the first- to fourth-quartile, or vice versa, between the first- and second-administered FFQ.

### 3.2. FFQ Validity

Correlations for the second-administered FFQ and mean DR varied considerably by subgroups defined by age group and child participation in FFQ completion (Table 3). Age-adjusted correlations among children ages 0–7 were highest for vitamin C (0.66), total fat (0.67), and lowest for retinol (−0.06). High correlations were observed in children 8–14 years who participated in FFQ administration with their caregiver (*n* = 23) or alone (*n* = 1). Calcium (0.54) and total folate (0.53) performed well, while nutrients such as total carbohydrates (−0.30) and zinc (−0.05) had lower correlations. Age-adjusted correlations among children 8–14 were substantially lower when the caregiver responded to the FFQ alone, with most ranging from −0.50–0.10. As observed in the reproducibility analysis, energy-adjustment decreased correlations for the majority of nutrients (data not shown). The percentage of participants in the FFQ classified into the same or adjacent quartile in the DR ranged from 61–77%; 5–11% of participants switched between extreme quartiles when comparing the FFQ and DR.

## 4. Discussion

We examined the validity of an FFQ to assess a wide range of micro- and macro-nutrients among children and adolescents in urban Peru. Results suggest that the tool was reproducible and performed well when compared with DRs in children under seven, the majority of which were completed by the caregiver alone, and in children ages 8–14 years who participated in the food frequency interview with their caregivers. While it may be reasonable for caregivers to complete the FFQ on behalf of their younger children, involving older children and adolescents in FFQ completion was critical to achieving valid results in our study.

The reproducibility results from our study are comparable to the few studies in children from other settings. The correlations in our study were similar to those of an FFQ administered among elementary and high school students in Puerto Rico (mean 0.22) [24]. Other studies have reported correlations as high as 0.50 to 0.70, though these studies were among adolescents ages 12–19 years who completed questionnaires without caregiver assistance [25,26,27]. The modest reproducibility coefficients in our study may be the result of the longer test-retest period (six months), which included multiple seasons [6]. While administering an FFQ at a short interval may result in respondents remembering their responses, administering an FFQ at lengthy intervals can result in the assessment of true changes in dietary habit [28].

Correlations for validity between the FFQ and DRs were similar to those reported in other pediatric studies. A systematic review of micronutrient assessment in children and adolescents found most coefficients in the range of 0.20 to 0.60 [8]. Similar to our study, studies reporting a large variety of nutrients reported low correlations (<0.20) for some nutrients alongside high correlations for others (>0.50), evidencing the tendency for validity coefficients to vary widely within a study [29,30,31]. Low correlations in our study suggest that our FFQ may not be able to accurately assess intake of nutrients that did not perform well, such as iron, retinol, and folate. Low correlations (<0.10) between the FFQ and DR for children 8–14 years who did not participate in the FFQ interview may not necessarily reflect the inability of the caregiver to accurately recall their child’s intake. Rather, this finding may be explained by different levels of child involvement between the DR and FFQ, or an increased frequency of eating meals outside of the home in this group, necessitating that older children and adolescents participate in questionnaire completion.

Participants in the validation study were recruited from a cohort study of household contacts of tuberculosis patients, which took place in urban and predominantly low-income areas of Lima. Tuberculosis patients in Peru may receive nutritional counseling during treatment. The extent to which this limited counseling affects diet, particularly in low income areas where limited financial resources may constrain food purchasing choices, is unknown. If a household member of a study participant began treatment during our study period and changed their diet, it is theoretically possible that this may have affected the household’s diet. The result of this could be reduced FFQ reproducibility and possibly lower correlations between the DR and FFQ. Additionally, one child developed tuberculosis midway through the study period. Though the effect is likely small, reproducibility and validity may have been affected if this participant’s diet substantially changed due to illness.

A number of factors may have negatively impacted the validity statistics in our study. First, due to logistical constraints, we used different nutrient databases to calculate intake from DRs and the FFQ. Second, the length of our 150-item FFQ may have had an effect on its validity. One review of validation studies in children and adolescents found that 20–60 item questionnaires were associated with higher validity [6]; however, other reviews have suggested there is no effect of questionnaire length on validity [32]. FFQ validity is highly dependent on local diet. Because our study setting was urban and largely low-income, care should be taken to include any relevant adaptations prior to applying this FFQ to cohorts outside of Lima or in wealthier populations within Lima. Quartile agreement in our study was reasonable indicating there was minimal extreme misclassification. Nonetheless, increasing the sample size to account for misclassification may be warranted in future applications of this FFQ.

Finally, we collected the three DRs over a six-month period covering multiple seasons. Diet in Peru can be subject to seasonal variation, typically resulting in higher caloric intake during the winter months, as was seen in our study [33,34]. In sensitivity analyses exploring seasonality, we observed only slightly higher correlations, suggesting that the influence of seasonality if present, is not a major source of bias.

## 5. Conclusions

In conclusion, we offer a valid FFQ that could prove helpful to understanding dietary risk factors for conditions affecting children and adolescents in urban Peru. Future work could examine whether self-administration by caregivers and/or children and abbreviating the questionnaire could further tool performance.

## Figures and Tables

**Table 1:** Characteristics of study participants at second-administered food frequency questionnaires (FFQ) (*N* = 118).

	Age 0–7 (*n* = 65)	Age 8–14 (*n* = 53)	Total (*N* = 118)
Age (years), mean ± SD	4.81 (1.86)	11.00 (2.03)	7.59 (3.64)
Male, *n* (%)	36 (55.38)	23 (43.40)	59 (50.0)
Weight (kg), mean ± SD	19.63 (6.15)	40.11 (11.28)	28.83 (13.49)
Height (cm), mean ± SD	104.21 (13.23)	141.22 (11.45)	120.83 (22.26)
BMI for age z-score, mean ± SD	1.08 (0.96)	0.62 (0.96)	0.87 (0.98)
Weight for age z-score, mean ± SD	0.41 (1.14)	0.26 (1.08)	0.49 (1.84)
Height for age z-score, mean ± SD	−0.54 (0.94)	−0.45 (0.95)	−0.26 (2.73)
Second-administered FFQ completed by			
Caregiver alone	62 (95.38)	29 (54.72)	91 (77.12)
Caregiver and child	3 (4.62)	23 (43.40)	26 (22.03)
Child alone	0	1 (1.89)	1 (0.85)

Abbreviations: Standard deviation (SD), body mass index (BMI).

**Table 2:** Reproducibility of log-transformed energy and nutrients from food frequency questionnaires (FFQs) administered at month 1 (FFQ1) and month 6 (FFQ2) (*N* = 120).

Nutrient (Unit)	FFQ1, Mean ± SD	FFQ2, Mean ± SD ^a^	FFQ1/ FFQ2, % ^a^	Same or Adjacent Quartile, % ^a^	Extreme Quartile, % ^a^	Pearson Correlation Coefficient ^a^		
						Age 0–7 (*n* = 66)	Age 8–14 (*n* = 52)	All (*n* = 118)
Energy (kcal)	8.06 (0.34)	7.93 (0.25)	101.7	73.5	6.8	0.29	0.26	0.28
Cholesterol (mg)	6.25 (0.47)	5.98 (0.38)	104.4	70.9	9.4	0.37	0.08	0.25
**Macronutrients**								
*Plasma fatty acids*								
Total Monounsaturated Fatty Acids (g)	3.53 (0.39)	3.31 (0.30)	106.6	76.9	6.8	0.27	0.39	0.31
Total Polyunsaturated Fatty Acids (g)	3.36 (0.38)	3.21 (0.31)	104.8	73.5	8.5	0.22	0.11	0.17
*Other macronutrients*								
Animal Protein (g)	4.42 (0.41)	4.22 (0.32)	104.7	68.4	6.8	0.28	0.24	0.26
Total Carbohydrate (g)	6.07 (0.36)	5.96 (0.29)	101.8	70.1	5.1	0.26	0.21	0.24
Total Dietary Fiber (g)	3.60 (0.40)	3.42 (0.36)	105.2	68.4	6.0	0.26	0.15	0.22
Total Fat (g)	4.67 (0.37)	4.47 (0.29)	104.3	76.9	6.8	0.26	0.36	0.30
Total Protein (g)	4.85 (0.36)	4.69 (0.27)	103.4	66.7	6.0	0.29	0.09	0.22
**Micronutrients**								
*Carotenoids*								
α-Carotene (μg)	7.46 (0.61)	7.22 (0.71)	103.2	73.5	8.5	0.21	0.26	0.23
β-Carotene (μg)	9.05 (0.54)	8.77 (0.58)	103.3	69.2	7.7	0.25	0.35	0.27
β-Cryptoxanthin (μg)	6.61 (0.75)	6.27 (0.81)	105.5	72.6	11.1	0.31	0.23	0.28
*Tocopherols*								
β-Tocopherol (mg)	−0.38 (0.47)	−0.57 (0.40)	67.4	76.9	6.0	0.29	0.28	0.29
δ-Tocopherol (mg)	2.03 (0.43)	1.87 (0.40)	108.3	65.8	9.4	0.08	0.04	0.06
γ-Tocopherol (mg)	3.27 (0.41)	3.11 (0.38)	105.0	66.7	10.3	0.12	0.04	0.08
*Other micronutrients*								
Calcium (mg)	7.11 (0.38)	6.93 (0.35)	102.6	66.7	6.8	0.28	0.10	0.20
Iron (mg)	3.16 (0.45)	3.02 (0.36)	104.7	67.5	5.1	0.24	0.12	0.18
Niacin (vitamin B3) (mg)	3.64 (0.38)	3.51 (0.30)	103.7	71.8	6.0	0.33	0.11	0.28
Retinol (μg)	6.68 (0.64)	6.28 (0.66)	106.4	67.5	7.7	0.44	−0.02	0.23
Riboflavin (vitamin B2) (mg)	0.99 (0.36)	0.76 (0.32)	130.3	65.8	8.5	0.30	−0.01	0.18
Total Folate (μg)	6.47 (0.37)	6.31 (0.32)	102.5	65.0	4.3	0.35	0.09	0.27
Total Vitamin A Activity (IU)	9.89 (0.48)	9.59 (0.50)	103.1	75.2	7.7	0.28	0.35	0.29
Total Vitamin A Activity (RAE) (μg)	7.45 (0.47)	7.11 (0.48)	104.8	70.1	6.0	0.37	0.21	0.31
Total Vitamin A Activity (RE) (μg)	7.86 (0.46)	7.54 (0.47)	104.2	74.4	7.7	0.33	0.29	0.31
Vitamin C (mg)	5.48 (0.50)	5.23 (0.50)	104.8	60.7	10.3	0.38	0.16	0.31
Vitamin D (μg)	2.08 (0.54)	1.78 (0.30)	117.1	75.2	9.4	0.31	0.08	0.22
Vitamin E (mg)	2.57 (0.40)	2.32 (0.32)	110.8	70.9	6.0	0.33	0.21	0.28
Zinc (mg)	2.72 (0.37)	2.58 (0.30)	105.5	64.1	6.0	0.28	0.07	0.19

^a^ *n* = 118. Abbreviations: standard deviation (SD), milligram (mg), microgram (μg), gram (g), international units (IU), kilocalories (kcal), retinol activity equivalents (RAE), retinol equivalents (RE).

**Table 3:** Quartile classification and crude and age-adjusted correlation coefficients of select nutrients observed in the second-administered FFQ and mean 24-h diet recall.

Nutrient	Same or Adjacent Quartile, % (*N* = 118)	Extreme Quartile, % (*N* = 118)	Age-Adjusted Correlation Coefficient			
			Age 0–7 (*n* = 65)	Age 8–14 (*n* = 53)		All (*N* = 118)
				Caregiver and Child, Child alone (*n* = 24)	Caregiver Alone (*n* = 29)	
Energy	67.80	7.63	0.46	0.12	0.30	0.33
**Macronutrients**						
Animal Protein	72.03	9.32	0.35	0.09	0.07	0.24
Total Carbohydrate	65.25	6.78	0.36	−0.30	0.28	0.26
Total Dietary Fiber	65.52	8.62	0.56	0.14	−0.62	0.33
Total Fat	77.12	5.08	0.67	0.51	0.59	0.54
Total Protein	70.34	10.17	0.30	0.16	−0.23	0.17
Vegetable Protein	66.95	7.63	0.35	0.09	−0.39	0.17
**Micronutrients**						
Calcium	72.88	6.78	0.41	0.54	0.35	0.37
Iron	61.02	11.02	0.18	0.10	−0.53	0.00
Niacin (vitamin B_3_)	68.64	8.47	0.27	0.54	−0.35	0.20
Retinol	66.10	9.32	−0.06	0.41	0.02	0.03
Riboflavin (vitamin B_2_)	69.49	6.78	0.18	0.13	0.20	0.16
Total Folate	63.56	11.02	0.16	0.53	−0.55	0.06
Vitamin C (ascorbic acid)	65.25	6.78	0.66	0.11	0.02	0.40
Zinc	71.19	10.17	0.44	-0.05	−0.10	0.22

## Supplementary Materials

The following are available online at www.mdpi.com/2072-6643/9/10/1121/s1, Table S1: Means and standard deviations of select untransformed nutrients from the FFQ, Table S2: Comparison of non-adjusted and energy adjusted Pearson correlation coefficients in reproducibility analysis of log-transformed nutrients from first- and second-administered food frequency questionnaires (FFQ) (*N* = 118).
