# Supplementary material for: Development and Validation of a Food Frequency Questionnaire to Estimate Intake among Children and Adolescents in Urban Peru
Source: Nutrients. 2017 Oct 14;9(10):1121. doi: 10.3390/nu9101121 (PMC5691737; doi:10.3390/nu9101121)
Supplement: Supplementary file 1 [file nutrients-09-01121-s001.docx]

Table S1: Means and standard deviations of select untransformed nutrients from the FFQ

| Nutrient | First-administered  FFQ (n=120),  mean ± SD | Second-administered  FFQ (N=118),  mean ± SD |
| --- | --- | --- |
| Energy (kcal) | 3376.2 (1379.4) | 2853.0 (725.7) |
| **Macronutrients** |  |  |
| *Plasma fatty acids* |  |  |
| Total Monounsaturated Fatty Acids (g) | 36.8 (16.0) | 28.6 (8.8) |
| Total Polyunsaturated Fatty Acids (g) | 31.0 (12.4) | 25.9 (8.5) |
| *Other macronutrients* |  |  |
| Animal Protein (g) | 90.4 (43.7) | 71.5 (24.1) |
| Cholesterol (mg) | 577.9 (308.7) | 425.6 (160.2) |
| Total Carbohydrate (g) | 466.2 (218.3) | 404.8 (118.2) |
| Total Dietary Fiber (g) | 39.9 (20.9) | 32.6 (12.8) |
| Total Fat (g) | 113.9 (46.5) | 91.3 (27.5) |
| Total Protein (g) | 136.8 (59.0) | 113.0 (32.0) |
| **Micronutrients** |  |  |
| *Carotenoids* |  |  |
| α-Carotene (mcg) | 2090.2 (1501.6) | 1626.2 (848.1) |
| β-Carotene (mcg) | 9977.4 (6932.6) | 7421.4 (4211.2) |
| β-Cryptoxanthin (mcg) | 960.6 (816.7) | 691.8 (489.0) |
| *Tocopherols* |  |  |
| β-Tocopherol (mg) | 0.8 (0.4) | 0.6 (0.3) |
| δ-Tocopherol (mg) | 8.4 (3.8) | 7.0 (2.9) |
| γ-Tocopherol (mg) | 28.7 (12.5) | 24.1 (9.2) |
| *Other micronutrients* |  |  |
| Calcium (mg) | 1323.4 (605.1) | 1084.7 (375.3) |
| Iron (mg) | 26.7 (17.5) | 21.9 (8.9) |
| Niacin (vitamin B3) (mg) | 41.2 (18.2) | 35.1 (11.1) |
| Retinol (mcg) | 965.6 (616.0) | 654.8 (416.7) |
| Riboflavin (vitamin B2) (mg) | 2.9 (1.3) | 2.2 (0.7) |
| Total Folate (mcg) | 699.6 (357.4) | 579.3 (188.1) |
| Total Vitamin A Activity (IU) | 22388.6 (13799.4) | 16481.0 (8377.8) |
| Total Vitamin A Activity (RAE) (mcg) | 1924.9 (1068.5) | 1369.8 (663.8) |
| Total Vitamin A Activity (RE) (mcg) | 2882.8 (1648.1) | 2084.8 (1005.1) |
| Vitamin C (mg) | 271.1 (166.7) | 208.6 (97.2) |
| Vitamin D (mcg) | 9.4 (6.8) | 6.7 (3.9) |
| Vitamin E (mg) | 14.4 (7.9) | 10.8 (3.7) |
| Zinc (mg) | 16.5 (7.9) | 13.8 (4.3) |

**Abbreviations:** food frequency questionnaire (FFQ), standard deviation (SD), milligram (mg), microgram (mcg), gram (g), international units (IU), kilocalories (kcal), retinol activity equivalents (RAE), retinol equivalents (RE)

Table S2. Comparison of non-adjusted and energy adjusted Pearson correlation coefficients in reproducibility analysis of log-transformed nutrients from first- and second- administered food frequency questionnaires (FFQ) (N=118)

| Nutrient | Pearson correlation coefficient |  | Energy-adjusted Pearson correlation coefficient |
| --- | --- | --- | --- |
| Energy (kcal) | 0.28 |  | |
| Cholesterol (mg) | 0.25 | 0.20 | |
| **Macronutrients** |  |  | |
| *Plasma fatty acids* |  |  | |
| Total Monounsaturated Fatty Acids | 0.31 | 0.13 | |
| Total Polyunsaturated Fatty Acids | 0.17 | -0.04 | |
| *Other macronutrients* |  |  | |
| Animal Protein | 0.26 | 0.17 | |
| Total Carbohydrate | 0.24 | 0.02 | |
| Total Dietary Fiber | 0.22 | 0.14 | |
| Total Fat | 0.30 | 0.11 | |
| Total Protein | 0.22 | 0.05 | |
| **Micronutrients** |  |  | |
| *Carotenoids* |  |  | |
| α-Carotene | 0.23 | 0.19 | |
| β-Carotene | 0.27 | 0.21 | |
| β-Cryptoxanthin | 0.28 | 0.18 | |
| *Tocopherols* |  |  | |
| β-Tocopherol | 0.29 | 0.30 | |
| δ-Tocopherol | 0.06 | -0.06 | |
| γ-Tocopherol | 0.08 | -0.07 | |
| *Other micronutrients* |  |  | |
| Calcium | 0.20 | 0.11 | |
| Iron | 0.18 | 0.13 | |
| Niacin (vitamin B3) | 0.28 | 0.24 | |
| Retinol | 0.23 | 0.22 | |
| Riboflavin (vitamin B2) | 0.18 | -0.04 | |
| Total Folate | 0.27 | 0.11 | |
| Total Vitamin A Activity | 0.29 | 0.24 | |
| Total Vitamin A Activity (RAE) | 0.31 | 0.28 | |
| Total Vitamin A Activity (RE) | 0.31 | 0.27 | |
| Vitamin C | 0.31 | 0.20 | |
| Vitamin D | 0.22 | 0.16 | |
| Vitamin E | 0.28 | 0.16 | |
| Zinc | 0.19 | 0.03 | |

**Abbreviations:** food frequency questionnaire (FFQ), retinol activity equivalents (RAE), retinol equivalents (RE)
